# High Effectiveness in Actions of Carfilzomib on Delayed-Rectifier K^+^ Current and on Spontaneous Action Potentials

**DOI:** 10.3389/fphar.2019.01163

**Published:** 2019-10-07

**Authors:** Edmund Cheung So, Ping-Yen Liu, Chien-Ching Lee, Sheng-Nan Wu

**Affiliations:** ^1^Department of Anesthesia, An Nan Hospital, China Medical University, Tainan, Taiwan; ^2^Graduate Institute of Medical Sciences, Chang Jung Christian University, Tainan, Taiwan; ^3^Division of Cardiovascular Medicine, National Cheng Kung University Hospital, College of Medicine, National Cheng Kung University, Tainan, Taiwan; ^4^Institute of Imaging and Biomedical Photonics, National Chiao Tung University, Tainan, Taiwan; ^5^Department of Physiology, National Cheng Kung University Medical College, Tainan, Taiwan; ^6^Institute of Basic Medical Sciences, National Cheng Kung University Medical College, Tainan, Taiwan

**Keywords:** carfilzomib, delayed-rectifier K^+^ current, current inactivation, M-type K^+^ current, action potential, pituitary cell, vascular smooth muscle cell, heart cell

## Abstract

Carfilzomib (CFZ, Kyprolis^®^) is widely recognized as an irreversible inhibitor of proteasome activity; however, its actions on ion currents in electrically excitable cells are largely unresolved. The possible actions of CFZ on ionic currents and membrane potential in pituitary GH_3_, A7r5 vascular smooth muscle, and heart-derived H9c2 cells were extensively investigated in this study. The presence of CFZ suppressed the amplitude of delayed-rectifier K^+^ current (*I*_K(DR)_) in a time-, state-, and concentration-dependent manner in pituitary GH_3_ cells. Based on minimal reaction scheme, the value of dissociation constant for CFZ-induced open-channel block of *I*_K(DR)_ in these cells was 0.33 µM, which is similar to the IC_50_ value (0.32 µM) used for its efficacy on inhibition of *I*_K(DR)_ amplitude. Recovery from *I*_K(DR)_ block by CFZ (0.3 µM and 1 µM) could be well fitted by single exponential with 447 and 645 ms, respectively. The M-type K^+^ current, another type of K^+^ current elicited by low-threshold potential, was slightly suppressed by CFZ (1 µM). Under current-clamp condition, addition of CFZ depolarized GH_3_ cells, broadened the duration of action potentials as well as raised the firing frequency. In A7r5 vascular smooth muscle cells or H9c2 cardiac cells, the CFZ-induced inhibition of *I*_K(DR)_ remained efficacious. Therefore, our study led us to reflect that CFZ or other structurally similar compounds should somehow act on the activity of membrane K_V_ channels through which they influence the functional activities in different types of electrically excitable cells such as endocrine, neuroendocrine cells, smooth muscle cells, or heart cells, if similar *in vivo* findings occur.

## Introduction

Carfilzomib (CFZ, Kyprolis^®^), an analog of epoxomicin, is a second-generation irreversible tetrapeptide epoxyketone class proteasome inhibitor which is recognized as an anti-cancer drug. CFZ was known to target the chymotrypsin-like β5 subunit of the constitutive 20S proteasome or the β5i subunit of the immunoproteasome 20Si, along with minimal cross reactivity to other proteases (Zhou et al., 2009; Dou and Zonder, 2014; Kaplan et al., 2017). Predominantly through the inhibition of proteasome-mediated proteolysis, the presence of CFZ can induce cell cycle arrest and apoptosis in different types of human cancer cell lines including multiple myeloma, lymphoma, and various solid tumors (Demo et al., 2007; Zhang and Sidhu, 2014; Kaplan et al., 2017). Moreover, there were case reports on CFZ associated neurotoxicity (Kaplan et al., 2017; Karademir et al., 2018; Luczkowska et al., 2018). However, how this agent and other structurally similar compounds exert any perturbations on membrane ionic currents is not thoroughly investigated, although CFZ might manipulate the energy and redox metabolism through a mechanism independent of its inhibition of proteasome activity (Soriano et al., 2016).

Recently, acute administration of CFZ was shown to produce short-term modifications on vascular and myocardial properties such as myocardial ischemia, arterial hypertension, or acute renal dysfunction (Gavazzoni et al., 2018; Heckmann et al., 2018; Efentakis et al., 2019). They reported that cardiotoxicity of CFZ could have been linked to its action through inhibition of AMPKα phosphorylation and autophagy-related proteins (Efentakis et al., 2019). Furthermore, previous studies have demonstrated that proteasome inhibitors (e.g., PSI [*N*-[(phenylmethoxy)carbonyl]-L-isoleucyl-L-α-glutamyl-*tert*-butyl ester-*N*-[(1S)-1-formyl-3-methylbutyl]-L-alaninamide]) could induce apoptotic changes in pituitary tumor (GH_3_) cells, suggesting that these agents were thought to be potential novel agents for treating pituitary tumors not amenable to other treatments (Yu et al., 2002).

Here, we investigated the effects of CFZ on ionic currents in pituitary tumor (GH_3_) cells, A7r5 vascular smooth muscle cells and heart-derived H9c2 cells. The possible efficacy of CFZ on spontaneous action potentials (APs) in GH_3_ cells was also examined. Findings from our study revealed that CFZ can interact directly with K_V_ channels to alter the amplitude and gating (i.e., inactivation kinetics) of *I*_K(DR)_ in response to membrane depolarization.

## Materials and Methods

### Chemicals, Drugs and Solutions

Carfilzomib (CFZ, Kyprolis^®^, (αS)-α-[[2-(4-morpholinyl)acetyl]amino]benzenebutanoyl-L-leucyl-N-[(1S)-3-methyl-1-[[(2R)-2-methyl-2-oxiranyl]carbonyl]butyl]-L-phenylalaninamide, C_40_H_57_N_5_O_7_) was obtained from Cayman Chemical (Ann Arbor, MI), and ML-213 and 9-phenanthrol were from Tocris (Bristol, UK), while 4-aminopyridine, diazoxide, tetraethyl ammonium chloride and tetrodotoxin were from Sigma-Aldrich (St. Louis, MO). For cell preparations, all culture media, fetal bovine serum, horse serum, L-glutamine, trypsin/EDTA, penicillin-streptomycin, fungizone, and trypsin were obtained from Invitrogen (Carlsbad, CA). All other chemicals, such as CdCl_2_, HEPES and aspartic acid, were commercially available and of reagent grade. The water was de-ionized using a Milli-Q water purification system (Millipore, Bedford, MA).

The composition of the extracellular solution (i.e., HEPES-buffered normal Tyrode’s solution) was 136.5 mM NaCl, 5.4 mM KCl, 1.8 mM CaCl_2_, 0.53 mM MgCl_2_, 5.5 mM glucose, and 5.5 mM HEPES-NaOH buffer, pH 7.4. To measure whole-cell M-type K^+^ current (*I*_K(M)_), high K^+^-bathing solution was composed of the following: 145 mM KCl, 0.53 mM MgCl_2_, and 5 mM HEPES-KOH buffer, pH 7.4. To record either whole-cell *I*_K(DR)_ and *I*_K(M)_ or membrane potential, we filled the recording pipettes by using a solution consisting of 130 mM K-aspartate, 20 mM KCl, 1 mM KH_2_PO_4_, 1 mM MgCl_2_, 3 mM Na_2_ATP, 0.1 mM Na_2_GTP, 0.1 mM EGTA, and 5 mM HEPES-KOH buffer, pH 7.2. The pipette solution used was filtered on the day of use with a syringe filter with a 0.22 mm pore size (Millipore).

### Cell Preparations

GH_3_, a clonal cell line derived from a rat prolactin-secreting pituitary tumor, was obtained from the Bioresources Collection and Research Center (BCRC-60015; Hsinchu, Taiwan). Briefly, cells were cultured in Ham’s F-12 medium supplemented with 15% heat-inactivated horse serum (v/v), 2.5% fetal calf serum (v/v) and 2 mM L-glutamine in a humidified environment of 5% CO_2_/95% air (Wu et al., 2000; Wu et al., 2017). To promote cell differentiation, GH_3_ cells were transferred to a serum-free, Ca^2+^-free medium. Under our experimental conditions, cells remained 80–90% viable for at least two weeks.

The A7r5 cell line, derived from rat thoracic aortae, was obtained from American Type Culture Collection (ATCC; [CRL-1444], Manassas, VA), while the H9c2 cell line, derived from embryonic rat ventricles, also from ATCC ([CRL-1446]). A7r5 or H9c2 cells were maintained and subcultured in Dulbecco’s modified Eagle’s medium supplemented with heat-inactivated fetal bovine serum (10%), and 2 mM glutamine. They were grown in monolayer culture in 50 ml plastic culture flasks in a humidified environment of 5% CO_2_/95% air at 37 °C. The experiments were commonly performed after GH_3_, A7r5 or H9c2 cells reached confluence (usually 5–7 days).

### Electrophysiological Measurements and Recordings

Shortly before the electrical recordings, cells (e.g., GH_3_, A7r5, or H9c2 cells) were harvested and transferred to a home-made recording chamber positioned on the stage of an inverted microscope. Cells were immersed at room temperature (22–25 °C) in normal Tyrode’s solution, the composition of which was described above. Patch-clamp recordings under the whole-cell mode were achieved with either an RK-400 (Bio-Logic, Claix, France) or an Axopatch-200B amplifier (Molecular Devices, Sunnyvale, CA) (Wu et al., 2000; Wu et al., 2017). Patch electrodes with tip resistances of 3–5 MΩ were made of Kimax-51 borosilicate capillaries (#34500; Kimble, Vineland, NJ) on either a PP-830 puller (Narishige, Tokyo, Japan) or a P-97 horizontal puller (Sutter, Novato, CA), and then fire-polished with an MF-83 microforge (Narishige). The signals, comprising voltage and current tracings, were stored online at 10 kHz in an ASUSPRO-BU401LG computer (ASUS, Taipei City, Taiwan) controlled by pCLAMP 10.7 software (Molecular Devices). Changes in membrane potential recorded from GH_3_ cells were measured under current-clamp configuration. In a separate set of whole-cell *I*_K(DR)_ recordings with intracellular dialysis, the recording pipettes used were filled with the internal solution containing 0.3 µM CFZ.

### Data Analyses

To evaluate concentration-dependent inhibition of CFZ on the amplitude of *I*_K(DR)_, cells were immersed in Ca^2+^-free Tyrode’s solution. Each cell examined was maintained at −50 mV and 1−s depolarizing pulse from −50 to +50 mV was delivered. Current amplitudes at the level of +50 mV in response to depolarizing pulses were measured in the control and during cell exposure to different concentrations (0.01–10 µM) of CFZ. The concentration required to suppress 50% of current amplitude was determined by use of a Hill function:

$$\text{Relative amplitude}=\frac{\left(1-\text{a}\right)\times {\left[\text{C}\right]}^{-{\text{n}}_{\text{H}}}}{{\left[\text{C}\right]}^{-{\text{n}}_{\text{H}}}+{\text{IC}}_{50}^{-{\text{n}}_{\text{H}}}}+\text{a}$$

where [C] represents the CFZ concentration; IC_50_ and n_H_ are the concentration required for a 50% inhibition and the Hill coefficient, respectively; and maximal inhibition (i.e., 1−a) was also estimated from this equation. This equation converged reliably to produce the best-fit line and parameter estimates in **Figure 2D**.

To evaluate the steady-state inactivation curve of *I*_K(DR)_ during cell exposure to different CFZ concentrations, the relationships between the normalized amplitude of *I*_K(DR)_ and the conditioning potentials achieved in the presence of 0.3 or 1 µM CFZ were appropriately fitted with a Boltzmann function of the following form:

$$I=\frac{I_{max}}{1+\exp\left[\left(V-V_{\frac{1}{2}}\right)qF/RT\right]}$$

where *I*_max_ is the maximal peak amplitude of *I*_K(DR)_ taken at the level of +50 mV, *V*_1/2_ the voltage at which half-maximal inhibition occurs, *q* the apparent gating charge of the inactivation curve, *F* Faraday’s constant, *R* the universal gas constant, and *T* the absolute temperature.

### Statistical Analyses

Linear or nonlinear curve-fitting (e.g., sigmoidal or exponential fitting) to data sets shown in this work was achieved with least-squares minimization procedure by use of different maneuvers including Microsoft Solver function embedded in Excel (Microsoft) and 64-bit OriginPro 2016 program (OriginLab). The averaged results are presented as the mean ± standard error of the mean (SEM) with sample sizes (*n*) indicating the cell numbers from which the results were collected. The paired or unpaired Student’s *t*-test and a one-way analysis of variance (ANOVA) followed by post-hoc Fisher’s least-significance difference method, were implemented for statistical evaluation of differences among means. Assuming that normality underlying ANOVA was violated, we used non-parametric Kruskal–Wallis test. Statistical analyses were performed using the SPSS 20 statistical software package (IBM Corp., Armonk, New York). Statistical significance was determined at a *P*-value of <0.05.

## Results

### Effect of CFZ on Delayed-Rectifier K^+^ Current (*I*_K(DR)_) in GH_3_ Cells

**Figure 1:**
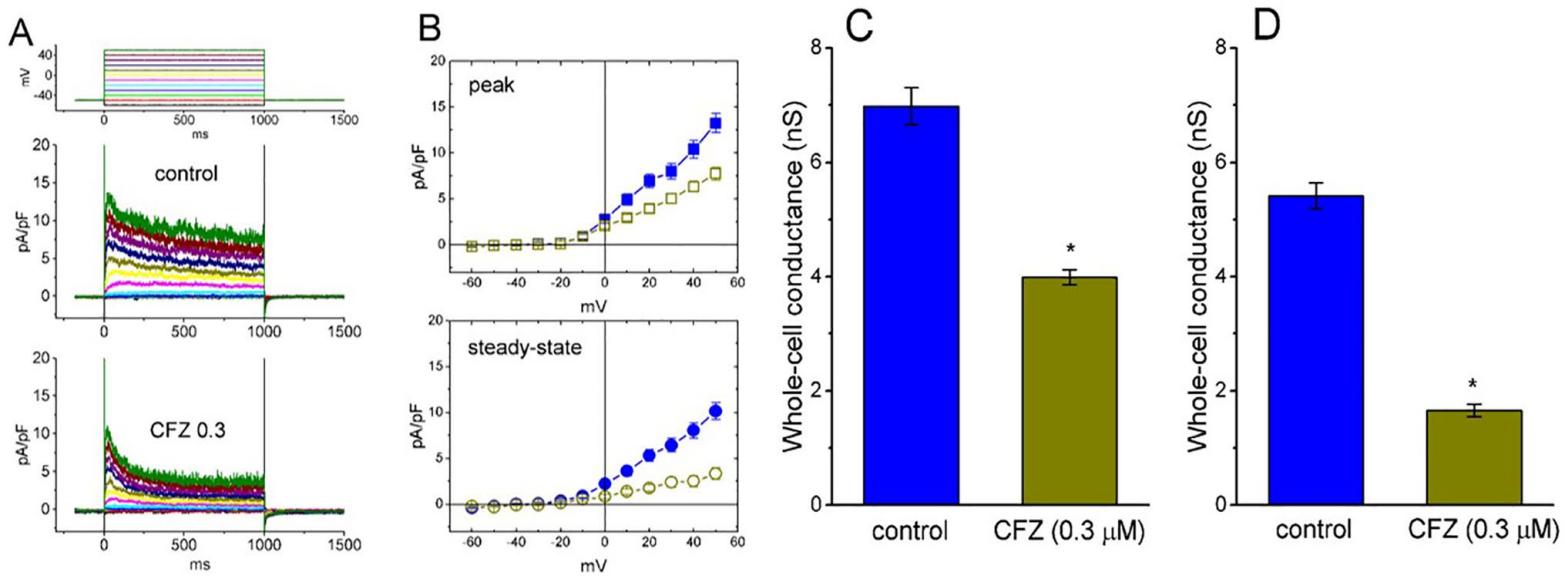
Effect of CFZ on *I*_K(DR)_ density in GH_3_ cells. In these whole-cell current recordings, cells were immersed in Ca^2+^-free Tyrode’s solution and the recording pipette was filled with K^+^-containing solution. The examined cells were held at −50 mV and different voltage pulses ranging from −60 to +50 mV in 10-mV increments were applied. **(A)** Representative traces for *I*_K(DR)_ density taken from the absence (upper; control) and presence (lower; CFZ 0.3) of 0.3 μM CFZ. The upper part in **(A)** indicates the voltage protocol applied. **(B)** Averaged current density versus voltage relationships of *I*_K(DR)_ measured at the beginning (filled symbols; upper [peak]) and end (open symbols; lower [steady-state]) of each depolarizing pulse. The data points in the upper (peak) and lower (steady-state) panels were obtained from the absence (square symbols) and presence (circle symbols) of 0.3 μM CFZ, respectively. Each data point represents mean ± SEM (n = 9). Summary bar graphs in **(C)** and **(D)** respectively show the whole-cell conductances of *I*_K(DR)_ at the beginning (i.e., peak conductance) and end (i.e., steady-state conductance) of depolarizing pulses ranging between 0 and +50 mV (mean ± SEM; n = 8 for each bar). *Significantly different from controls (*P* < 0.05).

The whole-cell configuration of the patch-clamp technique was employed to investigate any modifications of CFZ on ionic currents inherently in GH_3_ cells. In an initial set of experiments, to measure *I*_K(DR)_ and to avoid contamination of other types of ionic currents such as voltage-gated Na^+^ and Ca^+^ currents, and Ca^2+^-activated K^+^ currents, we bathed cells in Ca^2+^-free Tyrode’s solution containing tetrodotoxin (1 µM) and CdCl_2_ (0.5 mM), and, during electrophysiological recordings, we filled the recording pipette by using K^+^-containing solution. The compositions of these solutions used are detailed in *Materials and Methods*. As shown in **Figures 1A, B**, when the examined cell was maintained at −50 mV and a series of voltage pulses ranging from −60 to +50 mV in 10-mV increments were thereafter delivered, a family of K^+^ outward currents with slight inactivation was readily elicited. These outward K^+^ currents in response to membrane depolarization were previously proofed as the delayed-rectifier K^+^ current (*I*_K(DR)_) (Wu et al., 2000; Wang et al., 2008b). As cells were exposed to 1 mM tetraethyl ammonium chloride, a blocker of *I*_K(DR)_, the amplitude of *I*_K(DR)_ elicited by membrane depolarization from −50 to +50 mV was decreased to 86 ± 9 pA (n = 9, *P* < 0.05) from a control value of 282 ± 22 pA. However, the presence of 1 mM 4-aminopyridine, a blocker of transient outward K^+^ current, did not alter the amplitude of *I*_K(DR)_ (282 ± 22 pA [in the absence of 4-aminopyridine] versus 281 ± 24 pA [in the presence of 4-aminopyridine; n = 11, *P* > 0.05]). Of particular interest, during exposure of cells to CFZ (0.3 µM for 1 min), the amplitude of *I*_K(DR)_ measured at the end of 1−s depolarizing pulse was significantly reduced at the potentials ranging from 0 to +50 mV. For example, upon membrane depolarization from −50 to +50 mV, the presence of CFZ (0.3 µM) significantly decreased current density at the end of the voltage pulse from 10.1 ± 0.9 to 3.3 ± 0.6 pA/pF (n = 9, *P* < 0.05). This inhibitory effect was reversed to 7.6 ± 0.7 pA/pF (n = 7, *P* < 0.05) on washout of CFZ. Similarly, as cells were exposed to 0.3 µM CFZ, the whole-cell *I*_K(DR)_ conductances (at the peak or steady-state level) measured at the voltages ranging between 0 and +50 mV were reduced (**Figures 1C, D**). However, as intracellular dialysis with 0.3 µM CFZ, the *I*_K(DR)_ amplitude at the end of depolarizing pulse from −50 to +50 mV was not altered (356 ± 27 pA [in control] versus 354 ± 28 pA [in the presence of 0.3 µM CFZ]; n = 7, *P* > 0.05). Moreover, the activation kinetics of *I*_K(DR)_ (i.e., maximal d*I*/d*t* value in the rising phase of the current by membrane depolarization from −50 to +50 mV) did not differ between the absence and presence of 0.3 µM CFZ (26.1 ± 3.2 pA/ms [in control] versus 26.3 ± 3.4 pA/ms [in the presence of 0.3 µM CFZ]; n = 9, *P* > 0.05).

Of note, an increase in the decaying time course of *I*_K(DR)_ in response to sustained membrane depolarization was conceivably observed in the presence of CFZ (**Figure 1A**). These currents obtained after addition of CFZ activated a maximum and then rapidly decayed over time to a small fraction of their peak values seen at the voltages between 0 and +50 mV. However, in the presence of CFZ, there was no significant change in the activation kinetics (i.e., *dI*/*dt*) of these currents elicited by membrane depolarization. Averaged *I–V* relations for the amplitude of initial and steady-state components of *I*_K(DR)_ in the control and during the exposure to CFZ (0.3 µM) are shown in **Figure 1B**, respectively, indicating that the presence of CFZ diminished the *I*_K(DR)_ amplitude in a time-dependent manner, because of the increased relaxation in inactivation time course of *I*_K(DR)_.

### Kinetic Study of CFZ-Induced Block of *I*_K(DR)_ in GH_3_ Cells

**Figure 2:**
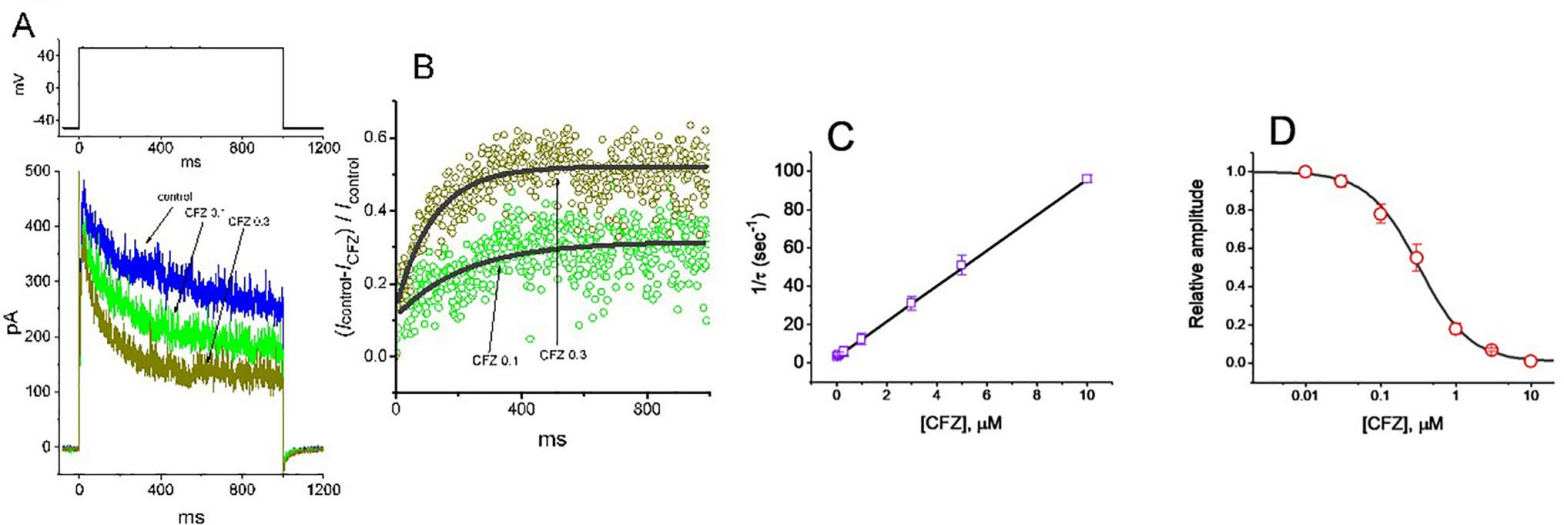
Evaluation of the kinetics of CFZ-induced block of *I*_K(DR)_ elicited by membrane depolarization in GH_3_ cells. The *I*_K(DR)_ established by depolarizing pulse from −50 to +50 mV with a duration of 1 s (indicated in the upper part of (A)) was measured as cells were exposed to different CFZ concentrations. In **(A)**, representative *I*_K(DR)_ traces was obtained in the absence (control) and presence of 0.1 μM CFZ (CFZ 0.1) or 0.3 μM CFZ (CFZ 0.3). In **(B)**, the time courses of relative block of *I*_K(DR)_ by 0.1 μM CFZ (CFZ 0.1) and 0.3 μM CFZ (CFZ 0.3) were well fitted by single exponential with a value of 181 and 91 ms (indicated by the smooth curves). The relative block (i.e., (*I*_control−_ *I*_CFZ_)/*I*_control_) was evaluated by dividing the CFZ-sensitive current by the current taken from the control (i.e., in the absence of CFZ). In **(C)**, the reciprocal of time constant (i.e., 1/τ) of relative block versus the CFZ concentration was plotted. Data points appearing in open squares were fitted by a linear regression, indicating that there is a molecularity of one. Mean ± SEM (n = 9 for each point). **(D)** Concentration-dependent inhibition of *I*_K(DR)_ by CFZ (mean ± SEM; n = 9 for each point). Each cell was maintained at −50 mV and the depolarizing pulses from −50 to +50 mV with a duration of 1 s were applied. Current amplitudes measured at the end of depolarizing pulses in the presence of different CFZ concentrations were compared with the control value. The smooth curve was appropriately fitted by the Hill function detailed in *Materials and Methods*. The IC_50_ value obtained in the presence of CFZ was calculated to be 0.32 µM.

To provide quantitative estimate for CFZ-induced block of *I*_K(DR)_, we further analyzed the time-dependent trajectories indicating relative block of *I*_K(DR)_ (i.e., (*I*_control−_ *I*_CFZ_)/*I*_control_) observed in these cells. We then fitted the time courses of relative block in the presence of different CFZ concentrations by a single-exponential function. The concentration dependence of *I*_K(DR)_ decay elicited by long-lasting membrane depolarization was shown in **Figures 2A, B**. The results clearly showed that with the presence of CFZ, it produced a concentration-dependent raise in the rate (1/τ) of relative block. For example, as cells were depolarized from –50 to +50 mV with a duration of 1 s, the time constants (τ) of *I*_K(DR)_ relative block obtained in the presence of 0.1, 0.3 and 1 µM CFZ were least-squares fitted by single exponential with the values of 173 ± 12, 88 ± 9, and 38 ± 7 ms (n = 9), respectively.

The inhibitory action of CFZ on *I*_K(DR)_ measured from GH_3_ cells is explained by state-dependent block that preferentially binds to the open state of the channel. A minimal kinetic scheme was derived as the following:


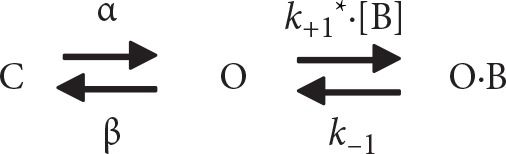


where [B] is the CFZ concentration; α and β are the voltage-gated rate constants for the opening and closing of K_V_ channels, respectively; *k*_+1_* and *k*_−1_ those for blocking and unblocking produced by the presence of CFZ, respectively; and C, O, and OˑB shown in the scheme represent the closed, open, and open-blocked states, respectively.

The blocking (i.e., on) and unblocking (i.e., off) rate constants, *k*_+1_* and *k*_−1_, were determined from time constants (τ) of depolarization-elicited relative block (i.e., (*I*_control−_ *I*_CFZ_)/*I*_control_) of *I*_K(DR)_ (Caballero et al., 2004) obtained in different concentrations (0.01–5 µM) of CFZ. These rate constants (1/τ) were then allowed to be computed using the relation (**Figure 2C**):

$$\frac{1}{\tau }=k_{+1}\times [B]+k_{-1}$$

where *k*_+1_* and *k*_−1_ are respectively acquired from the slope and from the y-axis intercept at [B] = 0 of the linear regression in which the reciprocal time constants (1/τ) versus the CFZ concentrations were interpolated, and [B] indicates the CFZ concentration.

Specifically, on the basis of the first-order reaction scheme, the relationship between 1/τ and [B] became linear with a correlation coefficient of 0.97 (**Figure 2C**). The blocking and unblocking rate constants were calculated to be 9.278 s^−1^µM^−1^ and 3.018 s^−1^, respectively; consequently, the results gave the value of dissociation constant (*K*_D_ = *k*_1_/*k*_+1_*) of 0.33 µM.

### Effect of CFZ on the Steady-State Inactivation Curve of *I*_K(DR)_ in GH_3_ Cells

**Figure 3:**
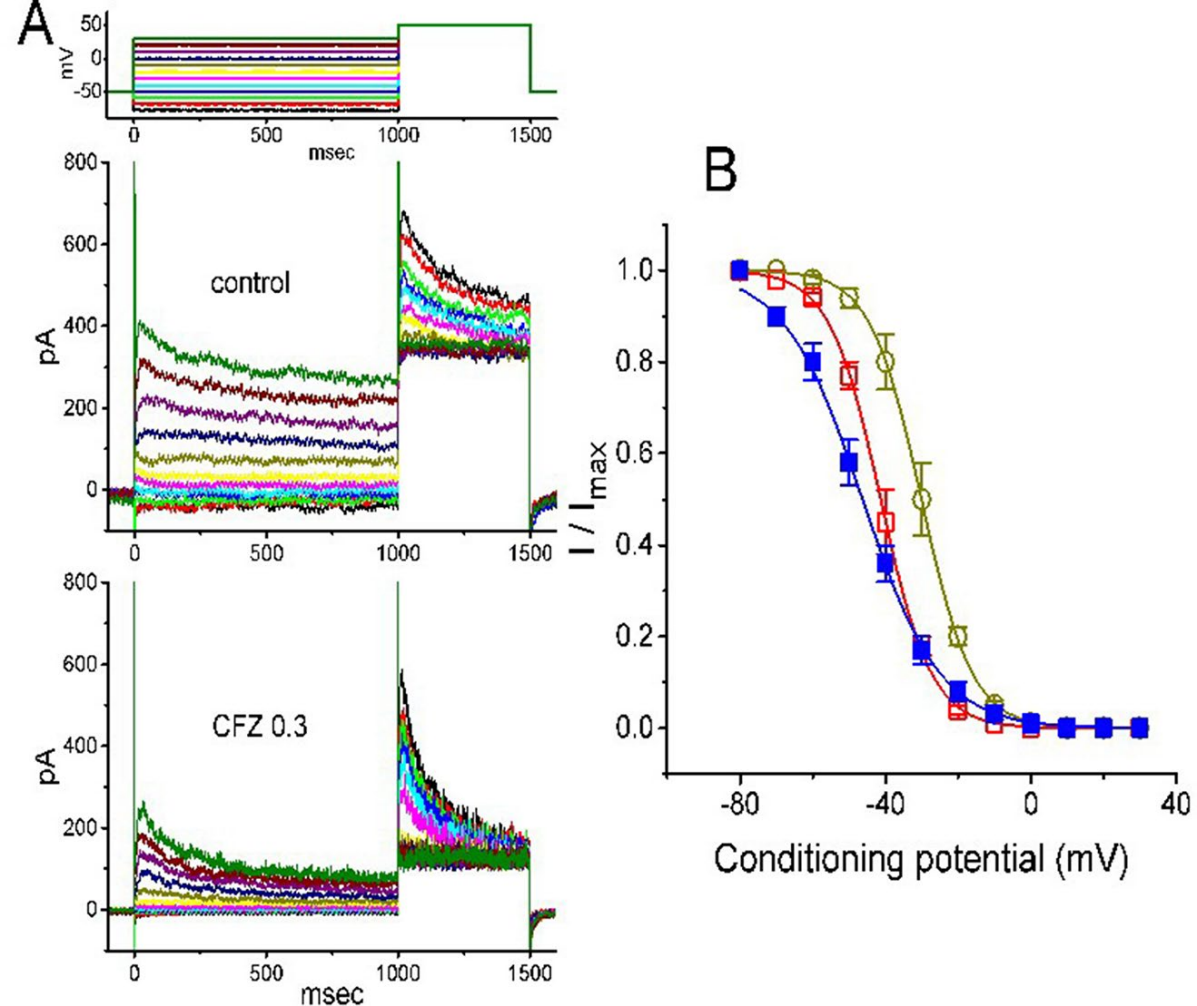
Effect of CFZ on the steady-state inactivation of *I*_K(DR)_ in GH_3_ cells. **(A)** Representative *I*_K(DR)_ traces obtained in the absence (upper) and presence (lower) of 0.3 µM CFZ. As the examined cell was held at −50 mV, the conditioning voltage pulses with a duration of 1 s to various membrane potentials ranging from −80 to +30 mV were delivered. Following each condition pulse applied, a test pulse to +50 mV with a duration of 500 ms was then applied to elicit *I*_K(DR)_. The upper part indicates the voltage protocol used. **(B)** Steady-state inactivation curve of *I*_K(DR)_ in the absence (filled squares) and presence of 0.3 µM (open circles) and 1 µM (open squares) CFZ (mean ± SEM; n = 8 for each point). The smooth curves were least-squares fitted by a Boltzmann function (detailed under *Materials and Methods*).

The steady-state inactivation of *I*_K(DR)_ during cell exposure to CFZ was further studied. In this set of experiments, cells were bathed in Ca^2+^-free Tyrode’s solution and the electrode was filled with K^+^-containing solution. As whole-cell configuration was firmly established, we applied a two-pulse protocol to the examined cells which were exposed to different CFZ concentrations. The inactivation parameters of *I*_K(DR)_ were then measured in the absence and presence of CFZ (1 or 3 µM). As illustrated in **Figure 3**, the normalized amplitudes of *I*_K(DR)_ (i.e., *I*/*I*_max_) was constructed against the conditioning potential and the smooth curve was fitted by a Boltzmann function described under *Materials and Methods*. In control (i.e., in the absence of CFZ), *V*_1/2_ = −46.3 ± 2.3 mV and *q* = 2.4 ± 0.3 *e* (n = 8). In the presence of 0.3 µM CFZ, *V*_1/2_ = −30.1 ± 2.2 mV and *q* = 3.6 ± 0.2 *e* (n = 8), while in the presence of 1 µM CFZ, *V*_1/2_ = −41.2 ± 2.1 mV and *q* = 3.6 ± 0.2 *e* (n = 8). Results from the above experiments showed that during the exposure to different CFZ concentrations, the inactivation curve of *I*_K(DR)_ measured from GH_3_ cells could be altered.

### Recovery From *I*_K(DR)_ Block Induced by CFZ

**Figure 4:**
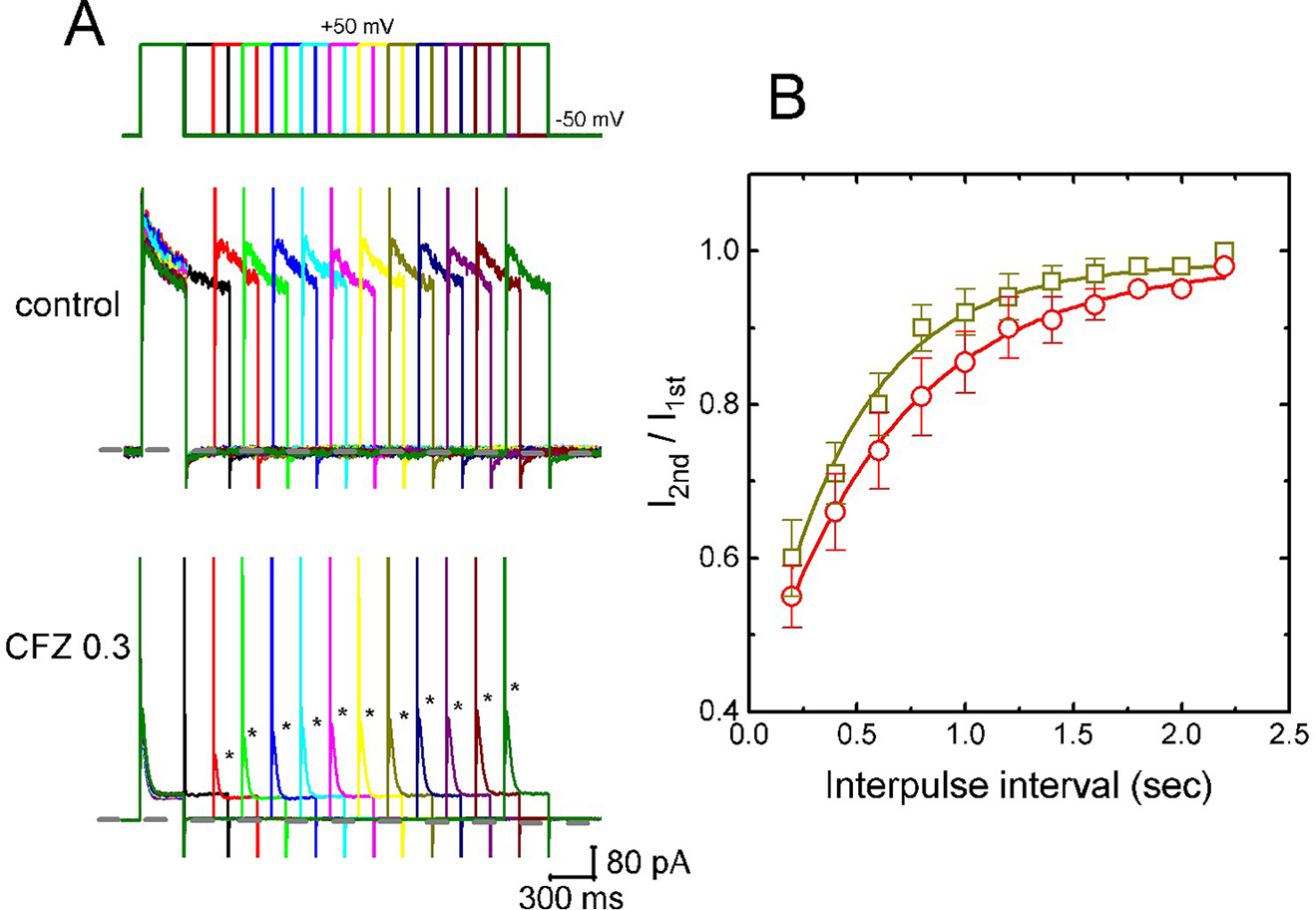
Time course of the recovery of *I*_K(DR)_ in the presence of 0.3 and 1 µM CFZ. In these experiments, GH_3_ cells, bathed in Ca^2+^-free Tyrode’s solution, were depolarized from −50 to +50 mV with a duration of 300 ms and different interpulse durations were applied. **(A)** Representative *I*_K(DR)_ traces in the absence (upper) and presence (lower) of 0.3 µM CFZ elicited by a two-pulse protocol with different interpulse durations (indicated in the upper part). Dashed line indicates the zero current level. **(B)** Time courses of recovery from *I*_K(DR)_ inactivation caused by 0.3 µM CFZ (open squares) and 1 µM CFZ (open circles), respectively. Smooth curves in the presence of different CFZ concentrations were fitted by single exponential. Each point represents the mean ± SEM (n = 8). Asterisks indicate the peak *I*_K(DR)_ elicited by different interpulse intervals.

In attempts to evaluate CFZ-induced block of *I*_K(DR)_ in GH_3_ cells, recovery from block was again investigated. In this set of experiments, a stimulation protocol consisted of a first (conditioning) depolarizing pulses which are sufficiently long to allow block to reach a steady state was used. While cells were exposed to CFZ (0.3 or 1 µM), the membrane potential was stepped to +50 mV from −50 mV for a variable time, after then a second depolarizing pulse (test pulse) was applied at the same potential as the conditioning pulse (**Figure 4A**). Then, the ratios of the peak *I*_K(DR)_ in response to the second (test) and first (conditioning) pulse were taken as a measure of recovery from block, and plotted versus interpulse interval. As shown in **Figure 4A**, the recovery of *I*_K(DR)_ was occurring so fast that the protocol used was not adequate to measure it. Hence, the time course for the recovery of *I*_K(DR)_ block was unable to be well illustrated. However, in our experimental protocol, *I*_K(DR)_ recovery during the exposure to 0.3 µM CFZ was generally complete, and its time course described by a single exponential with time constant of 447 ± 22 ms (n = 7). Moreover, the time constant of such recovery was significantly raised to 645 ± 25 ms (n = 7, *P* < 0.05) during cell exposure to 1 µM CFZ (**Figure 4B**), strongly suggesting that the slowing of *I*_K(DR)_ recovery produced by the presence of CFZ may result from open channel block.

### Effect of CFZ on M-Type K^+^ Current (*I*_K(M)_) in GH_3_ Cells

**Figure 5:**
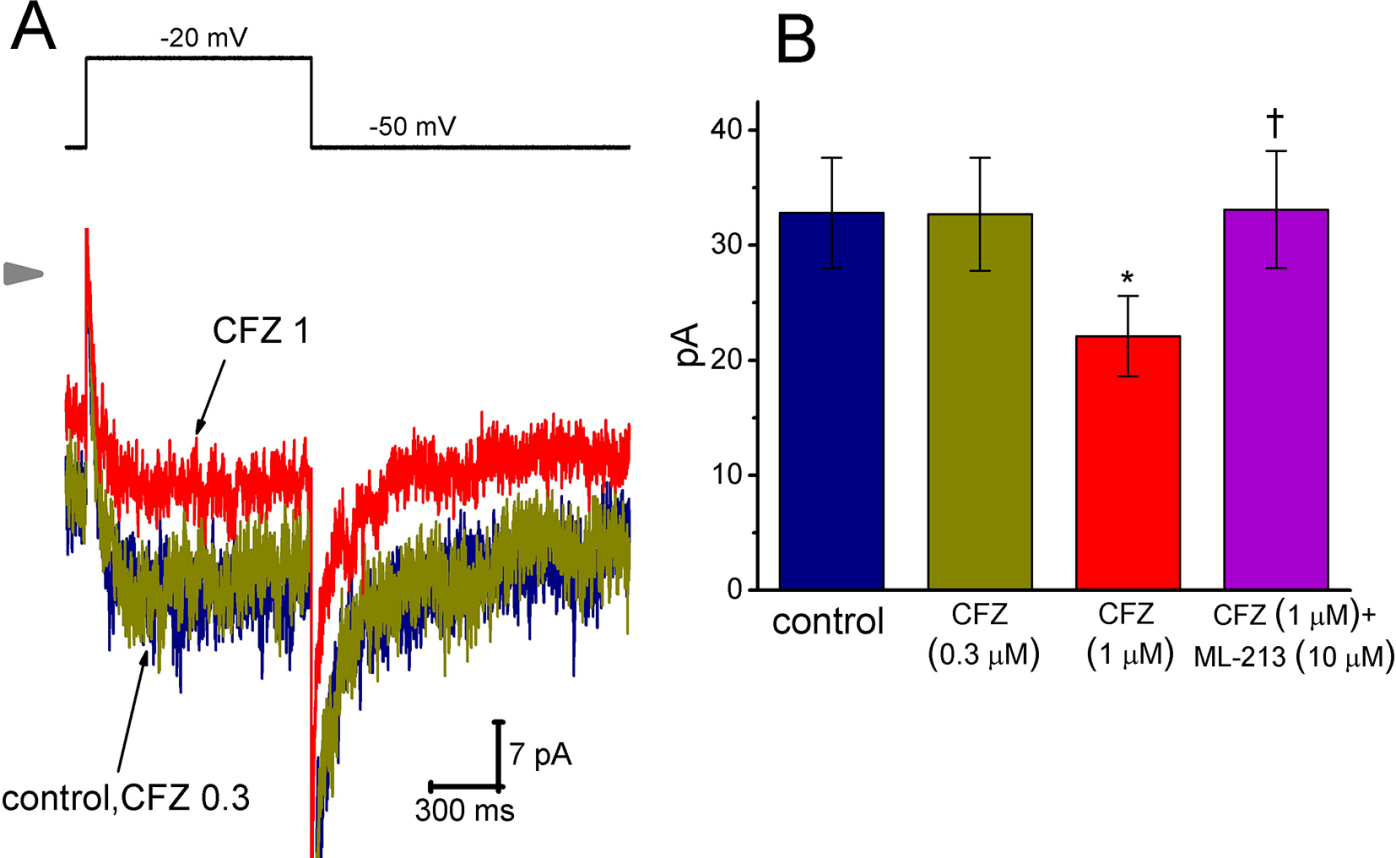
Effect of CFZ on *I*_K(M)_ in GH_3_ cells. To record *I*_K(M)_, we bathed cells in high-K^+^, Ca^2+^-free solution and filled the recording pipette by using K^+^-containing solution. **(A)** Representative *I*_K(M)_ traces in response to membrane depolarization (indicated in the upper part). CFZ 0.3:0.3 µM CFZ; CFZ 1:1 µM CFZ. Arrowhead shows the zero current level. **(B)** Bar graph showing the effects of CFZ and CFZ plus ML-213 on *I*_K(M)_ amplitude taken from GH_3_ cells (mean ± SEM; n = 8–9 for each bar). Current amplitude was measured at the end of depolarizing pulse from −50 to −20 mV. *Significantly different from control (*P* < 0.05) and ^†^significantly different from 1 µM CFZ alone group (*P* < 0.05).

Here, we further examined if addition of CFZ exerts any perturbations on other type of K^+^ current (i.e., *I*_K(M)_) inherently in GH_3_ cells. In this set of experiments, to amplify the magnitude of *I*_K(M)_ and preclude contamination of Ca^2+^-activated K^+^ currents, we bathed cells in high-K^+^, Ca^2+^-free solution and the recording pipettes used were filled with K^+^-containing solution (Chen et al., 2018; Hsiao et al., 2019; Liu et al., 2019; Lu et al., 2019). As cells were exposed to ML-213 (10 µM), an activator of *I*_K(M)_ (Yu et al., 2011), the amplitude of *I*_K(M)_ in response to membrane depolarization from -50 to −10 mV increased from 34 ± 11 to 57 ± 14 pA (n = 9, *P* < 0.05). As shown in **Figure 5**, cell exposure to 0.3 µM CFZ had minimal effect on *I*_K(M)_ elicited by membrane depolarization from −50 to −20 mV with a duration of 1 s. However, addition of 1 µM CFZ significantly diminished *I*_K(M)_ amplitude, as evidenced by a significant reduction of current density from 1312 ± 172 to 884 ± 153 fA/pF (n = 9, *P* < 0.05). Alternatively, in continued presence of 1 µM CFZ, further application of ML-213 (10 µM) significantly reversed *I*_K(M)_ density to 1324 ± 175 fA/pF (n = 8, *P* < 0.05). ML-213 was previously reported to stimulate *I*_K(M)_ (Yu et al., 2011). The results thus suggest that the *I*_K(M)_ in GH_3_ cells is relatively resistant to suppression by CFZ.

### Effect of CFZ on the Firing of Spontaneous Action Potentials (APs) in GH_3_ Cells

**Figure 6:**
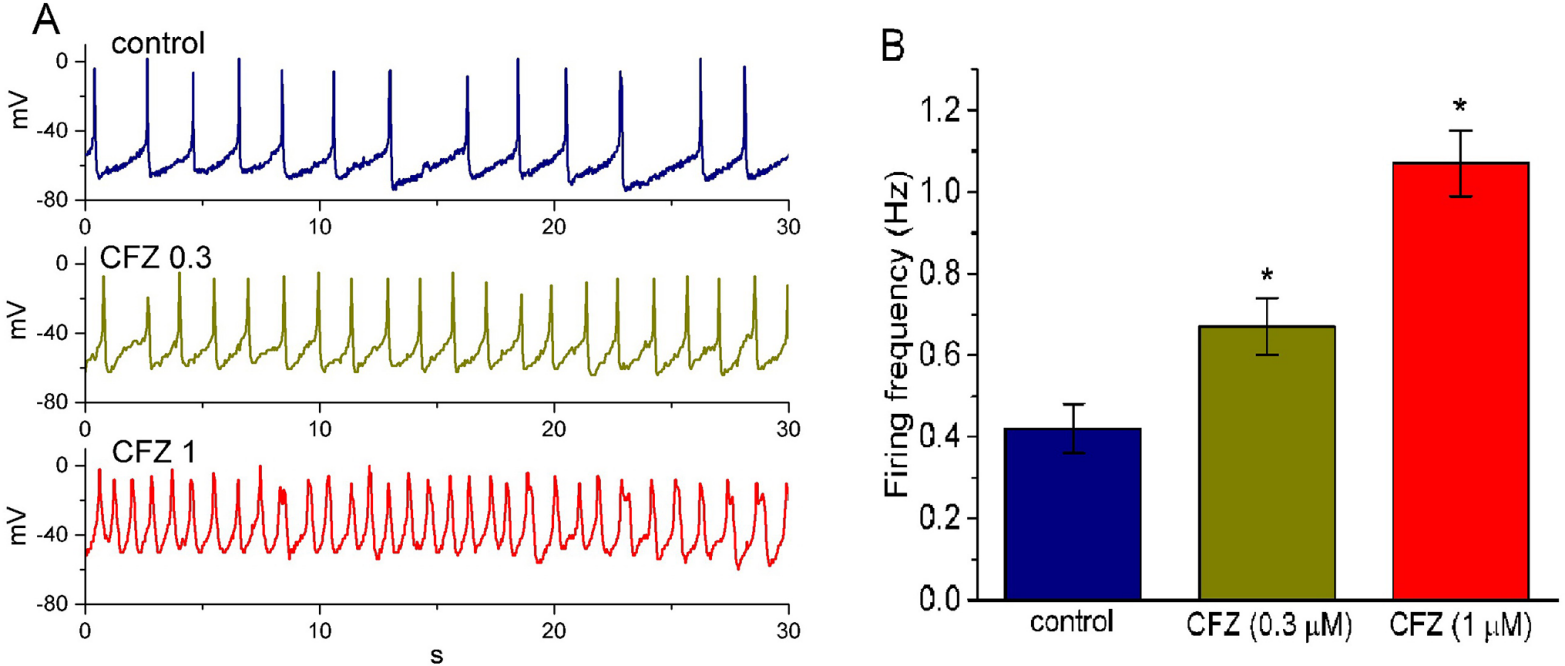
Effect of CFZ on spontaneous action potentials (APs) in GH_3_ cells. The experiments were conducted in cells bathed in normal Tyrode’s solution containing 1.8 mM CaCl_2_. As whole-cell configuration was achieved, the recording model was rapidly switched to current-clamp condition for measuring any changes in membrane potential. **(A)** Representative potential changes obtained in the absence (control) of CFZ and the presence of 0.3 µM CFZ (CFZ 0.3) and 1 µM CFZ (CFZ 1). **(B)** Bar graph depicting efficacy of CFZ to elevate the firing frequency of spontaneous APs in GH_3_ cells (mean ± SEM; n = 9 for each bar). *Significantly different from control (*P* < 0.05).

In another set of experiments, we switch the mode to current-clamp condition for measurement of any changes in membrane potential in these cells. During these potential recordings, cells were bathed in normal Tyrode’s solution containing 1.8 mM CaCl_2_ and the pipette was filled with K^+^-containing solution. As shown in **Figure 6**, the presence of CFZ produced a progressive increase in the firing frequency of APs. For example, CFZ at a concentration of 0.3 and 1 µM significantly raised AP frequency to 0.67 ± 0.07 and 1.07 ± 0.08 Hz (n = 9, *P* < 0.05), respectively, from a control value of 0.42 ± 0.06 Hz (n = 9). Concomitant with these, during the exposure to 0.3 and 1 µM CFZ, the cells was significantly depolarized to −61 ± 2 and −48 ± 2 mV (n = 9, *P* < 0.05), respectively, from a control value of −66 ± 3 mV (n = 9). The duration of APs also became widened in the presence of 1 µM CFZ, as evidenced by a significant prolongation of AP duration from 45 ± 9 to 155 ± 14 ms (n = 9, *P* < 0.05). Therefore, it is conceivable from these results that CFZ-induced changes in membrane potential tend to be closely connected to its modification of *I*_K(DR)_ amplitude and gating in these cells.

### Inhibitory Effect of CFZ on *I*_K(DR)_ Measured From A7r5 Vascular Smooth Muscle Cells and in Heart-Derived H9c2 Cells

**Figure 7:**
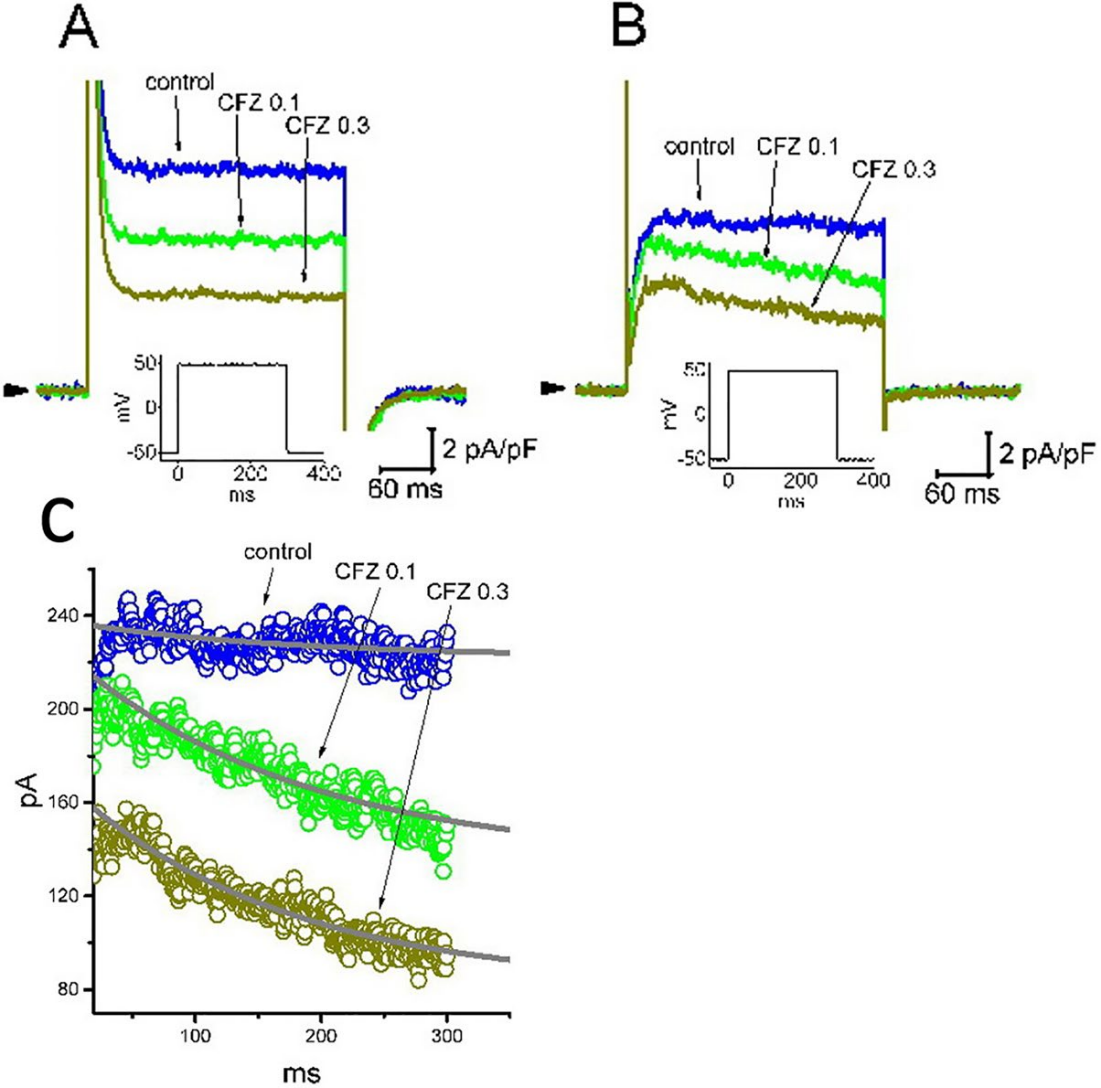
Inhibitory effect of CFZ on *I*_K(DR)_ in A7r5 vascular smooth muscle cells **(A)** and in H9c2 cardiac cells **(B)**. In these experiments, cells were bathed in Ca^2+^-free Tyrode’s solution and the pipette was filled with K^+^-containing solution. Superimposed *I*_K(DR)_ traces recorded from either an A7r5 cell **(A)** or an H9c2 cell **(B)** were respectively obtained in the absence (control) and presence of 0.1 µM CFZ (CFZ 0.1) and 0.3 µ M CFZ (CFZ 0.3). Arrowhead indicates the zero current level, and inset shown in each panel denotes the voltage protocol applied. **(C)** Inactivation time course of *I*_K(DR)_ in the absence and presence of 0.1 or 0.3 µM CFZ measured from H9c2 cells. Smooth curves labeled in control, CFZ 0.1 or CFZ 0.3 were least-squares fitted by single exponential.

Recent studies have recently demonstrated that CFZ might produce cardiovascular adverse events, although it evidently improves survival of patients suffering from multiple myeloma (Gavazzoni et al., 2018; Heckmann et al., 2018; Iannaccone et al., 2018; Mangla et al., 2018; Efentakis et al., 2019). For these reasons, we further evaluated whether addition of CFZ is able to exert any perturbations on *I*_K(DR)_ inherently in A7r5 vascular smooth myocytes or H9c2 cardiac cells. In these experiments, A7r5 or H9c2 cells were bathed in Ca^2+^-free Tyrode’s solution and the *I*_K(DR)_ was elicited when the examined cell was depolarized from −50 to +50 mV with a duration of 300 ms. As illustrated in **Figure 7**, the experimental results showed that CFZ at a concentration of 0.1 and 0.3 µM was effective at suppressing the *I*_K(DR)_ in these cells. For example, in A7r5 cells, the presence of CFZ (0.3 µM) decreased *I*_K(DR)_ density from 10.6 ± 2.1 to 4.4 ± 0.5 pA (n = 8, *P* < 0.05). However, distinguishable from its effect on GH_3_ or H9c2 cells, the presence of CFZ did not change the inactivation time course of *I*_K(DR)_ observed in A7r5 cells, despite its ability to suppress current amplitude effectively. Moreover, CFZ-mediated inhibition of *I*_K(DR)_ in A7r5 or H9c2 cells was unable to be attenuated by further addition of either diazoxide (30 µM), an activator of ATP-sensitive K^+^ currents, or 9-phenanthrol (3 µM), a stimulator of intermediate-conductance Ca^2+^-activated K^+^ channels (Garland et al., 2015; Chen et al., 2018). In A7r5 cells, the *I*_K(DR)_ density obtained in the presence of 0.3 µM CFZ and 0.3 µM CFZ plus 30 µM diazoxide did not differ significantly (4.4 ± 0.5 pA [CFZ alone] versus 4.3 ± 0.6 pA [CFZ plus diazoxide]; n = 8, *P* > 0.05). Similarly, current density in the presence of 0.3 µM CFZ or 0.3 µM CFZ plus 3 µM 9-phenanthrol was not different (4.4 ± 0.5 pA [CFZ alone] versus 4.4 ± 0.6 pA [CFZ plus 9-phenanthrol]; n = 8, *P* > 0.05). Moreover, in H9c2 cells, the presence of CFZ decreased *I*_K(DR)_ along with a significant reduction in inactivation time constant of the current (**Figure 7C**). The presence of CFZ at the concentration of 0.1 and 0.3 µM decreased the inactivation time constant of *I*_K(DR)_ to 185 ± 11 (n = 9, *P* < 0.05) and 171 ± 9 ms (n = 9, *P* < 0.05), respectively, from a control value of 196 ± 13 ms (n = 9).

## Discussion

The present results demonstrated that in pituitary tumor (GH_3_) cells, CFZ produced an inhibitory efficacy on delayed rectifier K^+^ current (*I*_K(DR)_) in a concentration- and state-dependent fashion. The main action of CFZ on *I*_K(DR)_ is thought to be principally through a state-dependent, open-channel block mechanism. Apart from its inhibitory effect on cytosolic proteasome or on changes in intracellular peptides (Dasgupta et al., 2014; Schäfer et al., 2017), block of CFZ of *I*_K(DR)_ presented herein is of peculiar importance, since it may have characteristics making it significant from both pathophysiological, therapeutic, or toxicological point of view.

In this study, the mechanism of CFZ action on *I*_K(DR)_ apparently differed from those on *I*_K(M)_. In pituitary tumor (GH_3_) cells, the effect exerted by the presence of CFZ is a time-, and concentration-dependent increase of the inactivation process of *I*_K(DR)_ elicited by long-lasting membrane depolarization, together with no change in the activation kinetics of the current. In other words, the inhibitory action of CFZ on *I*_K(DR)_ tends to correlate over time with a considerable raise in the inactivation rate of the currents in response to membrane depolarization. Additionally, CFZ at higher concentrations could suppress the amplitude of *I*_K(M)_ in GH_3_ cells. Both suppression of *I*_K(DR)_ and *I*_K(M)_ by CFZ might concertedly influence the electrical behavior of endocrine or neuroendocrine cells *in vivo* (Yu et al., 2002).

In light of steady-state inactivation curve of *I*_K(DR)_ during the exposure to CFZ, the voltage for half-maximal inactivation was noted to be in the range of AP firing. Additionally, cell exposure to CFZ was also found to slow down the recovery of *I*_K(DR)_ inactivation in GH_3_ cells. Therefore, it is conceivable that the CFZ molecule has a higher affinity toward the open-inactivated state of K_V_ channels than toward the closed or resting state of the channels in these cells. In this way, the magnitude of CFZ-induced block on *I*_K(DR)_ tends to be voltage-dependent, and the magnitude of its current inhibition on these currents can be synergistically influenced by different pre-existing level of resting potentials, the firing rate of APs, or the CFZ concentration applied, assuming that CFZ action in cells *in vivo* is the same as those on GH_3_ cells shown herein.

Earlier reports have shown the ability of CFZ to modify the energy and redox metabolism in neoplastic plasma cells or in heart cells (Al-Harbi, 2016; Soriano et al., 2016). However, in the present study, application of neither diazoxide nor 9-phenanthrol, in the presence of CFZ, attenuated its inhibitory efficacy on *I*_K(DR)_ in A7r5 cells. Diazoxide is an activator of ATP-sensitive K^+^ current, while 9-phenanthrol can enhance the activity of intermediate-conductance Ca^2+^-activated K^+^ channels (Garland et al., 2015; Chen et al., 2018), though it could also suppress Ca^2+^-activated nonselective current (Simard et al., 2012). Therefore, the observed effect of CFZ on whole-cell *I*_K(DR)_ enriched in A7r5 cells is not involved in the suppression of the activities derived from either ATP-sensitive or Ca^2+^-activated K^+^ channels. Moreover, in H9c2 cells, the presence of CFZ was able to inhibit *I*_K(DR)_ together with a decrease in inactivation time constant of the current. The inhibitory effect on the amplitude and gating of *I*_K(DR)_ caused by CFZ could be predominantly responsible for its interaction with macroscopic *I*_K(DR)_.

Under current-clamp potential recordings, the addition of CFZ was found to depolarize the cells, to widen AP duration, and to raise the firing frequency of spontaneous APs recorded from GH_3_ cells in a concentration-dependent fashion. It is thus most likely that CFZ-induced increase of *I*_K(DR)_ inactivation in endocrine or neuroendocrine cells may enhance a process of long-lasting facilitation or potentiation (Lin et al., 2008; Wang et al., 2008b; Wu et al., 2008; Kuo et al., 2018), which is conceivably associated with the toxicological actions of CFZ and other structurally similar compounds including variable forms of peripheral neuropathy (Cai et al., 2016; Kaplan et al., 2017; Karademir et al., 2018; Luczkowska et al., 2018).

The values of *K*_D_ (calculated from the first-order kinetic scheme) and IC_50_ (required for CFZ-mediated inhibition of *I*_K(DR)_) measured from GH_3_ cells were virtually indistinguishable (i.e., around 0.3 µM). These values are noted to be much lower than that used for PSI-mediated induction of apoptotic changes in GH_3_ cells (Yu et al., 2002). PSI, the structure of which is shared by CFZ, is another inhibitor of proteasome activity. The IC_50_ value for CFZ-induced suppression of cell viability in RPMI-8226 myeloma cells obtained during one-hour exposure was around 40 nM (Schäfer et al., 2017). Therefore, its effective concentration required for the inhibition of *I*_K(DR)_ in different types of neoplastic cells probably overlaps with that for the suppression of proteasome activity (Yu et al., 2002; Schäfer et al., 2017). Because the maximal plasma concentration of CFZ reached in the blood *in vivo* was reported to be around 1000–2000 ng/ml (i.e., 1.4–2.8 µM) (Maruyama et al., 2018), such unanticipated modifications of *I*_K(DR)_ amplitude and gating are expected to occur at a concentration achievable in humans.

Indeed, a previous study showed that berberine, an anti-diabetic herbal agent, could suppress *I*_K(DR)_ in human myeloma (RPMI-8226) cells and that its inhibitory effects could be associated with inhibitory efficacy on cell proliferation or apoptosis (Wu et al., 1998; Hsu et al., 2012). It is thus tempting to speculate that, apart from its inhibition of proteasome, the direct inhibitory effect of CFZ on the amplitude and gating of *I*_K(DR)_ contributes to its actions on myeloma cells. It remains to be further determined to what extent such blocking action is connected with CFZ-induced inhibition of proliferation in neoplastic cells such as myeloma, glioma or lung cancer cells (Demo et al., 2007; Baker et al., 2014; Booth et al., 2014; Zhang and Sidhu, 2014; Kaplan et al., 2017).

In this study, intracellular dialysis with CFZ (0.3 µM) had minimal changes in either the *I_K_*_(DR)_ amplitude or the time course of *I*_K(DR)_ inactivation. The results reflect that block by CFZ of *I*_K(DR)_ seen in GH_3_, A7r5 or H9c2 cells tends to be rapid in onset once the channel is open. However, intracellular application of CFZ had minimal effects on *I*_K(DR)_, thus ruling out an intracellular binding site for CFZ. Therefore, this binding site must be at the extracellular site in a location only accessible when the channel is in the open state. CFZ-mediated inhibition of *I*_K(DR)_ or *I*_K(M)_ presented here is direct and independent of its possible actions on the activity of cytosolic proteasomes or on any changes in intracellular peptide levels (Dasgupta et al., 2014; Schäfer et al., 2017). On the other hand, it would be interesting to explore whether the suppression by CFZ of proteasome activity would secondarily ascribes from its blocking of membranous *I*_K(DR)_ to some extent in different types of neoplastic cells.

Recently, it has been demonstrated that administration of CFZ is able to affect adverse cardiovascular events (Gavazzoni et al., 2018; Heckmann et al., 2018; Iannaccone et al., 2018; Mangla et al., 2018; Efentakis et al., 2019). Protein quality control inside the cell, with which proteasome inhibitors mainly interfere, is thought to be an important part of the molecular machinery of cardiomyocytes, endothelial cells, and probably all other members of the cardiac environment (Heckmann et al., 2018). Previous studies also reported that inactivation of AMPKα and downregulation of autophagy-related proteins occurred rapidly after CFZ administration (Efentakis et al., 2019). However, in the present study, addition of CFZ effectively suppressed *I*_K(DR)_ amplitude along with enhanced inactivation rate of the current in heart-derived H9c2 cells. In A7r5 vascular smooth muscle cells, the amplitude of *I*_K(DR)_ was also suppressed by CFZ. Earlier studies have notably demonstrated the ability of aconitine, a diterpenoid cardiotoxin, to exert *I*_K(DR)_ block in a concentration- and state-dependent fashion (Lin et al., 2008; Wu et al., 2008; Wang et al., 2008a; Wu et al., 2011). Aconitine was previously reported to induce apoptosis in H9c2 cardiac cells (Gao et al., 2018). Therefore, our findings lend the credence to the notion that the modifications by CFZ or aconitine of *I*_K(DR)_ amplitude or gating inherently in vascular smooth muscle and in heart cells is intimately linked to its action on variable forms of cardiovascular catastrophes (Iannaccone et al., 2018; Mangla et al., 2018; Efentakis et al., 2019).

The effect of CFZ on the inactivation time course of *I*_K(DR_ could be variable. Previous studies have demonstrated that the *I*_K(DR)_ present in GH_3_ cells could be mediated by several different subtypes of K_V_ channels, including K_V_1.2, K_V_1.4, K_V_1.5, K_V_2.1_,_ K_V_2.2, K_V_3.2, K_V_4.1 and K_V_5.2 (Wulfsen et al., 2000). To what extent CFZ action on the inactivation kinetics is specific for a unique subset of K_V_ channels remains to be further studied.

The present study provided evidence to show that interaction of CFZ with *I*_K(DR)_ in different types of cells may not be fully explained only by its inhibition on proteasome activity. Regardless of the mode of CFZ action, the K_V_ channels enriched in different types of electrically excitable cells are presumably an important target for the action of it or other structurally similar compounds. Findings from the present results also make a case for examining whether the mechanistic direction described herein for actions of CFZ or other structurally similar agents (e.g., bortezomib and ixazomib) (Zhou et al., 2009; Dou and Zonder, 2014) has pharmacological, toxicological and clinical relevance in humans.

## Data Availability Statement

The datasets generated for this study are available on request to the corresponding author.

## Author Contributions

ES: manuscript preparation and data collection. P-YL: research idea and reference collection. C-CL: manuscript proof reading and preparation of figures. S-NW: performing the patchclamp and manuscript preparation.

## Funding

This research was partly supported by a research fund (ANHRF107-13) from An Nan Hospital, China Medical University, granted to ES.

## Conflict of Interest

The authors declared that the research was conducted in the absence of any commercial or financial relationships that could be construed as a potential conflict of interest.
